# Data on the synthesis and characterization of two novel polydentate ligands possessing unsymmetrical NH–urea fragment

**DOI:** 10.1016/j.dib.2018.08.136

**Published:** 2018-08-31

**Authors:** Stanislava Todorova, Maria Atanassova, Vanya Kurteva

**Affiliations:** a Institute of Organic Chemistry with Centre of Phytochemistry, Bulgarian Academy of Sciences, Acad. G. Bonchev street, Block 9, 1113 Sofia, Bulgaria; b University of Chemical Technology and Metallurgy, Department of General and Inorganic Chemistry, 8 Kliment Okhridski blvd., 1756 Sofia, Bulgaria

## Abstract

The data represent the synthesis and structural characterization of two novel polydentate ligands possessing unsymmetrical NH-urea fragment: an open-chain substituted aromatic compound with unsymmetrical urea and secondary amine units (**S1**) and fused aryloxazinone with unsymmetrical urea fragment (**S2**). The data include the analyzed NMR spectra, turbo-spray mass spectra, melting points and R_f_-values. 1D and 2D NMR spectra are given in the article. The efficiency of the ligands as synergists in the isolation and separation of lantanoid ions is reported in rerence. [1].

**Specifications Table**

**Table:** 

Subject area	*Chemistry*
More specific subject area	*Organic polydentate ligands*
Type of data	*Scheme, figures (NMR spectra)*
How data was acquired	*NMR (1D and 2D), mass spectroscopy, TLC, m.p.*
Data format	*Analyzed*
Experimental factors	*The ligands were isolated by flash chromatography on silica gel and were then recrystallized*
Experimental features	*Synthesis and characterization of novel polydentate ligands*
Data source location	*Sofia, Bulgaria*
Data accessibility	*The data are available within this article and present a supplementary material to the article “M. Atanassova, S. Todorova, V. Kurteva, N. Todorova, Insights into the synergistic selectivity of 4f-ions implementing 4-acyl-5-pyrazolone and two new unsymmetrical NH-urea ring molecules in an ionic liquid, Sep. Purif. Technol.; In preparation”**[1]*.

**Value of the data**

• The 2D NMR data permit full assignment of the signals in the spectra of the novel compounds.
• The ligands offer unlimited possibilities for NH-derivatization.
• The data can be useful for comparison with similar structures.

## 1 Data

The data include synthetic details and characterization by 1D and 2D NMR spectra, mass spectrometry, TLC and m.p. of novel organic polydentate ligands.

## 2 Experimental design, materials and methods

### 2.1 General

All reagents were purchased from Aldrich, Merck and Fluka and were used without further purification. The deuterated chloroform was purchased from Deutero GmbH. Fluka silica gel (TLC-cards 60778 with fluorescent indicator 254 nm) were used for TLC chromatography and R_f_-values determination. Merck Silica gel 60 (0.040–0.063 mm) was used for flash chromatography purification of the products. The melting points were determined in capillary tubes on SRS MPA100 OptiMelt (Sunnyvale, CA, USA) automated melting point system. The NMR spectra were recorded on a Bruker Avance II+ 600 spectrometer (Rheinstetten, Germany) in CDCl_3_; the chemical shifts were quoted in ppm in δ-values against tetramethylsilane (TMS) as an internal standard and the coupling constants were calculated in Hz. The spectra were processed with Topspin 2.1 program. The low-resolution mass spectra were taken on a HP 5973 Mass Selective Detector, the turbo spray spectrum on API 150EX (AB/MAS Sciex).

### 2.2 Synthesis and characterization

**Scheme 1:**
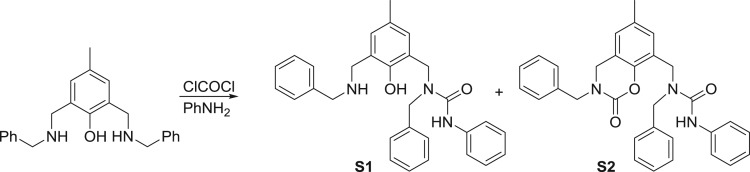
Synthesis of the ligands **S1** and **S2**.

Two novel polydentate ligands possessing unsymmetrical NH-urea fragment, **S1** and **S2** (Scheme 1) were synthesized as a mixture from a known bis-amine [2] and were isolated in low overall yield by flash chromatography on silica gel [3]. The optimization of protocols for each ligand is in progress.

To a solution of 2,6-bis((benzylamino)methyl)-4-methylphenol (3.92 mmol, 1.359 g), pyridine (12 mmol, 0.97 ml), and aniline (6 mmol, 0.55 ml) in toluene (20 ml) phosgene (8 mmol, 3.96 ml 20% solution in toluene) was added and the mixture was stirred at room temperature for 24 h. The products were partitioned between toluene and 10% aq. HCl. The water layer was washed with dichloromethane (DCM). The combined organic solutions were dried over MgSO_4_, evaporated to dryness, and purified by flash chromatography on silica gel by using mobile phase with a gradient of polarity from DCM to 2% MeOH in DCM.

Ligand **S1**: 13% (238 mg); R_f_ 0.22 (1% MeOH/DCM); m.p. 151.9–152.1 °C; ^1^H NMR 2.231 (s, 3H, C*H*_*3*_), 3.821 (s, 2H, C*H*_*2*_-Ph), 4.015 (s, 2H, C*H*_*2*_–6), 4.362 (s, 2H, C*H*_*2*_-2), 4.643 (s, 2H, C*H*_*2*_-Ph’), 6.800 (bs, 1H, C*H*-5), 6.843 (bs, 1H, C*H*-3), 6.979 (tt, 1H, J 7.4, 1.0, *p*-Ph”), 7.241 (dd, 2H, J 8.4, 7.5, *m*-Ph”), 7.268–7.328 (m, 6H, *o*-Ph, *m*-Ph, *p*-Ph, *p*-Ph’), 7.356 (dd, 2H, J 7.8, 7.4, *m*-Ph’), 7.398 (d, 2H, J 7.8, *o*-Ph’), 7.435 (bd, 2H, J 8.0, *o*-Ph”), 8.629 (bs, 1H, N*H*);^13^C NMR 20.49 (*C*H_3_), 45.21 (*C*H_2_-2), 49.20 (*C*H_2_-Ph’), 51.62 (*C*H_2_–6), 52.53 (*C*H_2_-Ph), 119.21 (*o*-Ph”), 121.93 (*C*_q_*-*6), 122.11 (*p*-Ph”), 123.51 (*C*_q_*-*2), 127.23 (*p*-Ph or *p*-Ph’), 127.84 (*p*-Ph or *p*-Ph’), 128.03 (*o*-Ph’), 128.43 (*o*-Ph), 128.57 (*C*_q_*-*4), 128.62 (*m*-Ph’), 128.73 (*m*-Ph”), 128.84 (*m*-Ph), 129.16 (*C*H-5), 130.79 (*C*H-3), 137.60 (*i*-Ph), 138.17 (*i*-Ph’), 140.26 (*i*-Ph”), 153.32 (*C*_q_*-*1), 156.42 (*C*=O-NH); ESI (TIS)-Q m/z 932 [2M+1]^+^ (28), 466 [M+1]^+^ (100), 359 [M-NHBn]^+^ (56), 347 [M-M-CHNHBn+1]^+^ (24), 240 [M-BnNCONHPh]^+^ (74), 226 [M-CH_2_N(Bn)CONHPh]^+^ (20), 133 [BnNCO]^+^ (41).

Ligand **S2**: 6% (120 mg); R_f_ 0.31 (1% MeOH/DCM); m.p. 173.7–174.4 °C; ^1^H NMR 2.258 (s, 3H, C*H*_*3*_), 4.279 (s, 2H, C*H*_*2*_–4), 4.595 (s, 2H, C*H*_*2*_–8), 4.652 (s, 2H, C*H*_*2*_-Ph’), 4.672 (s, 2H, C*H*_*2*_-Ph), 6.749 (bs, 1H, C*H*-5), 6.927 (bs, 1H, N*H*), 6.995 (tt, 1H, J 7.4, 1.1, *p*-Ph”), 7.015 (bs, 1H, C*H*-7), 7.255 (dd, 2H, J 8.4, 7.5, *m*-Ph”), 7.282–7.362 (m, 10H, *o*-Ph, *o*-Ph’, *m*-Ph, *m*-Ph’, *p*-Ph, *p*-Ph’), 7.406 (bd, 2H, J 8.1, *o*-Ph”);^13^C NMR NMR 20.80 (*C*H_3_), 45.33 (*C*H_2_–8), 46.56 (*C*H_2_-2), 50.43 (*C*H_2_-Ph’), 52.53 (*C*H_2_-Ph), 117.07 (*C*_q_*-*4a), 119.90 (*o*-Ph”), 122.82 (*p*-Ph”), 124.10 (*C*_q_*-*8), 125.33 (*C*H-5), 127.55 (*p*-Ph or *p*-Ph’), 127.59 (*p*-Ph or *p*-Ph’), 128.15 (*o*-Ph or *o*-Ph’), 128.24 (*o*-Ph or *o*-Ph’), 128.76 (*m*-Ph”), 128.84 (*m*-Ph or *m*-Ph’), 128.89 (*m*-Ph or *m*-Ph’), 129.30 (*C*H-7), 134.06 (*C*_q_*-*6), 135.19 (*i*-Ph), 137.35 (*i*-Ph’), 139.28 (*i*-Ph”), 145.41 (*C*_q_*-*8a), 150.50 (*C*_q_*-*2), 155.97 (*C*=O-NH); ESI (TIS)-Q m/z 983 [2M+1]^+^ (231), 492 [M+1]^+^ (100), 399 [M-NHPh]^+^ (9), 373 [M-CONPh+1]^+^ (42), 266 [M-BnNCONHPh]^+^ (30), 133 [BnNCO]^+^ (25).

### 2.3 Original NMR spectra

**Fig. 1:**
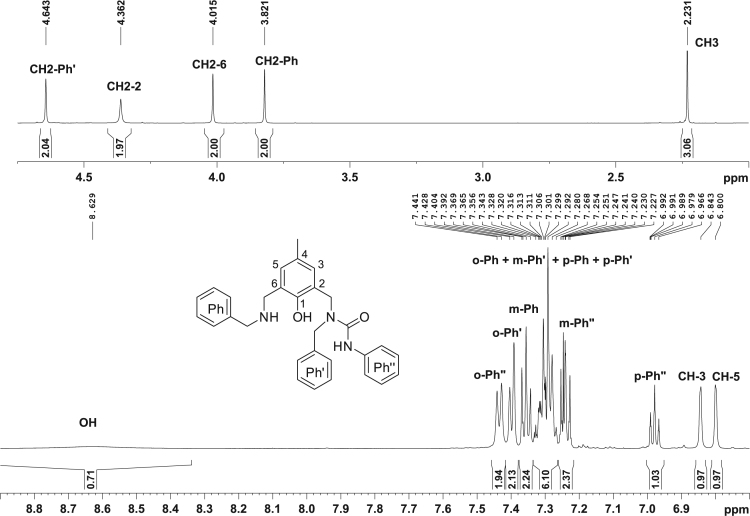
^1^H NMR spectrum of ligand **S1**.

**Fig. 2:**
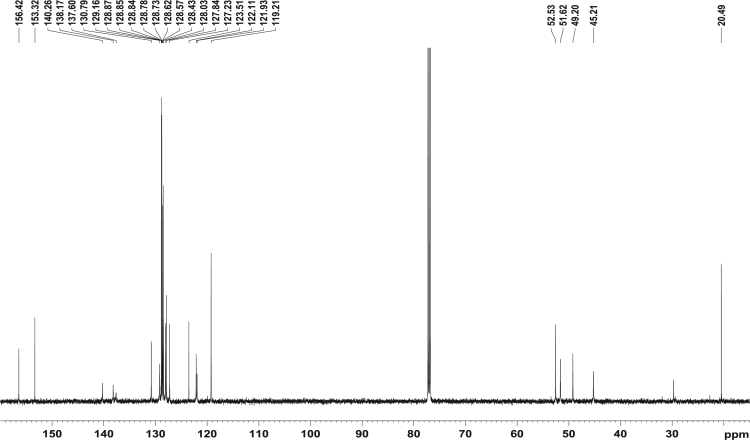
^13^C NMR spectrum of ligand **S1**.

**Fig. 3:**
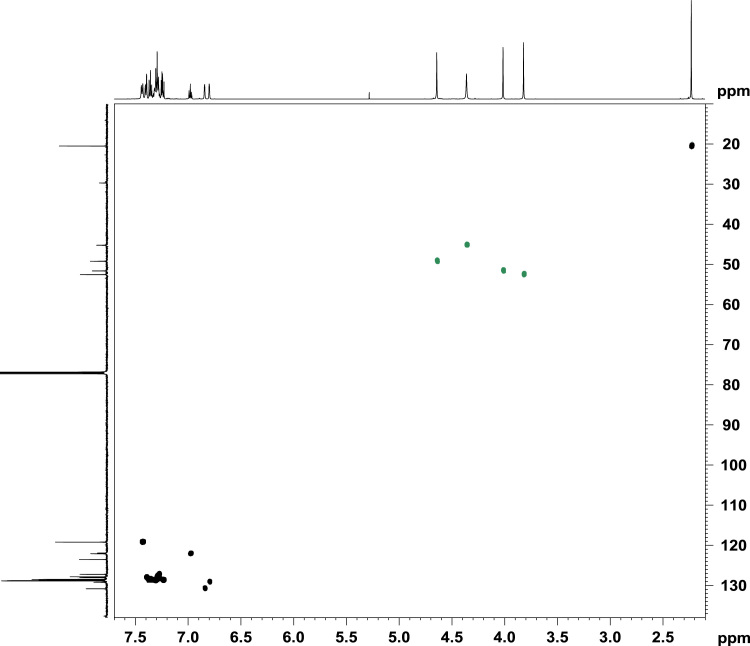
^1^H-^13^C HSQC spectrum of ligand **S1**.

**Fig. 4:**
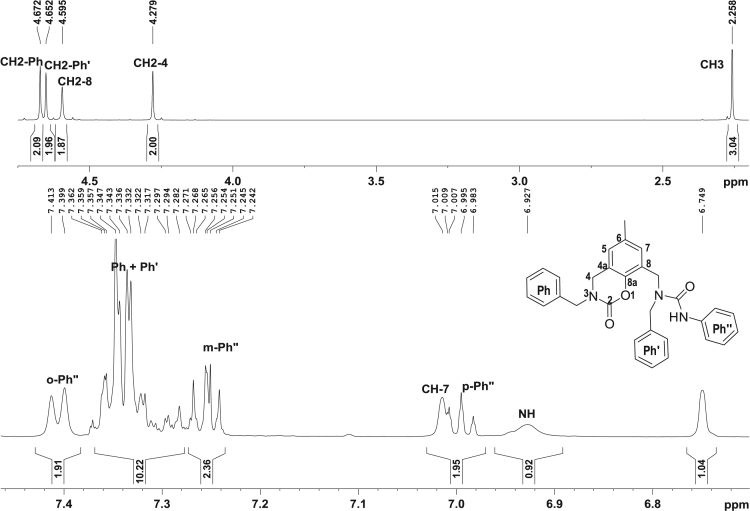
^1^H NMR spectrum of ligand **S2**.

**Fig. 5:**
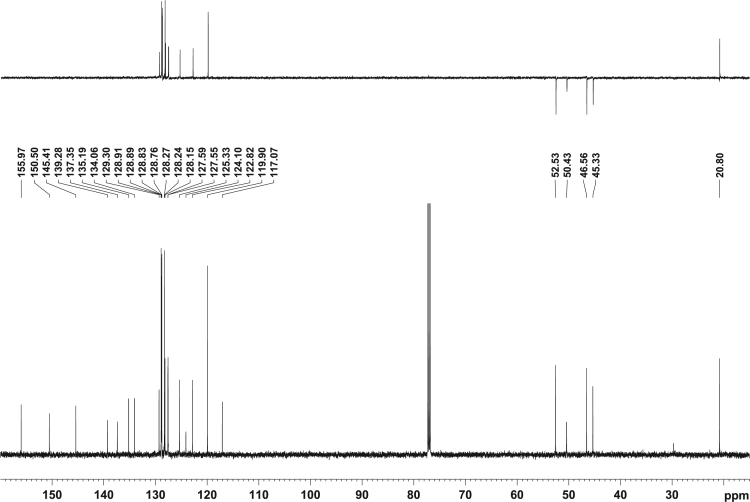
^13^C NMR spectrum of ligand **S2**.

**Fig. 6:**
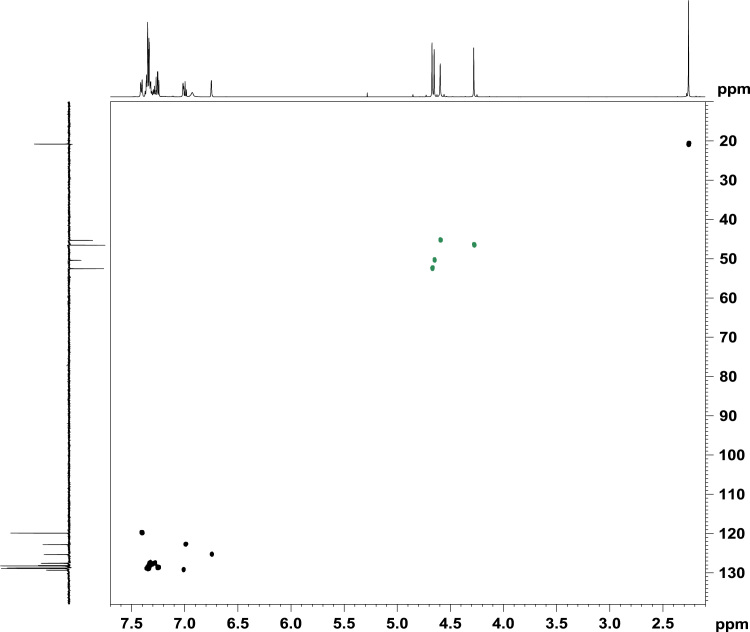
^1^H-^13^C HSQC spectrum of ligand **S2**.

See Fig. 1, Fig. 2, Fig. 3, Fig. 4, Fig. 5, Fig. 6.
