# Supplementary material for: Data on the synthesis and characterization of two novel polydentate ligands possessing unsymmetrical NH–urea fragment
Source: Data Brief. 2018 Aug 31;20:933–9. doi: 10.1016/j.dib.2018.08.136 (PMC6138974; doi:10.1016/j.dib.2018.08.136)
Supplement: Supplementary file 1 — Supplementary material [file mmc1.docx]

**Conflict of interest**

All the authors confirm no Conflict of interest.
